# Aldose reductase deficiency in mice protects from ragweed pollen extract (RWE)-induced allergic asthma

**DOI:** 10.1186/1465-9921-12-145

**Published:** 2011-11-03

**Authors:** Umesh CS Yadav, Leopoldo Aguilera-Aguirre, Istvan Boldogh, Kota V Ramana, Satish K Srivastava

**Affiliations:** 1 Departments of Biochemistry and Molecular Biology, 301 University Blvd., The University of Texas Medical Branch, Galveston, TX-77555, USA; 2 Microbiology and Immunology and Sealy Center for Molecular Medicine, 301 University Blvd., The University of Texas Medical Branch, Galveston, TX-77555, USA

**Keywords:** aldose reductase, allergic asthma, inflammation, ragweed pollen extract

## Abstract

**Background:**

Childhood hospitalization related to asthma remains at historically high levels, and its incidence is on the rise world-wide. Previously, we have demonstrated that aldose reductase (AR), a regulatory enzyme of polyol pathway, is a major mediator of allergen-induced asthma pathogenesis in mouse models. Here, using AR null (AR^-/-^) mice we have investigated the effect of AR deficiency on the pathogenesis of ragweed pollen extract (RWE)-induced allergic asthma in mice and also examined the efficacy of enteral administration of highly specific AR inhibitor, fidarestat.

**Methods:**

The wild type (WT) and AR^-/- ^mice were sensitized and challenged with RWE to induce allergic asthma. AR inhibitor, fidarestat was administered orally. Airway hyper-responsiveness was measured in unrestrained animals using whole body plethysmography. Mucin levels and Th2 cytokine in broncho-alveolar lavage (BAL) were determined using mouse anti-Muc5A/C ELISA kit and multiplex cytokine array, respectively. Eosinophils infiltration and goblet cells were assessed by H&E and periodic acid Schiff (PAS)-staining of formalin-fixed, paraffin-embedded lung sections. T regulatory cells were assessed in spleen derived CD4^+^CD25^+ ^T cells population.

**Results:**

Deficiency of AR in mice led to significantly decreased PENH, a marker of airway hyper-responsiveness, metaplasia of airway epithelial cells and mucus hyper-secretion following RWE-challenge. This was accompanied by a dramatic decrease in infiltration of eosinophils into sub-epithelium of lung as well as in BAL and release of Th2 cytokines in response to RWE-challenge of AR^-/- ^mice. Further, enteral administration of fidarestat significantly prevented eosinophils infiltration, airway hyper-responsiveness and also markedly increased population of T regulatory (CD4^+^CD25^+^FoxP3^+^) cells as compared to RWE-sensitized and challenged mice not treated with fidarestat.

**Conclusion:**

Our results using AR^-/- ^mice strongly suggest the role of AR in allergic asthma pathogenesis and effectiveness of oral administration of AR inhibitor in RWE-induced asthma in mice supports the use of AR inhibitors in the treatment of allergic asthma.

## Background

In spite of the identification of several factors associated with the development of allergic airway inflammation, a clear causative factor or mediator remains elusive. Asthma attacks are characterized by airway inflammation and narrowing leading to typical symptoms such as shortness of breath, cough, wheezing and chest tightness [1,2]. The attacks could be caused by varied stimuli such as allergens, including tree and grass pollens, dust mites, pet's hairs and dander, infections, exercise, sudden weather change, and environmental pollutants and irritant such as tobacco smoke [3,4]. The severity could vary from mild to life-threatening attacks. Although asthma develops at different stages of life, it is one of the leading chronic childhood disease (9.3% prevalence) and major cause of disability in children in the United States [5]. Based on the 2008 NHIS sample, it was estimated that 38.4 million Americans, or 128.5 per 1,000 persons, had been diagnosed with asthma by a health professional within their lifetime [5].

Multiple interacting risk factors such as allergens, environmental tobacco smoke, particulate matter, oxides of nitrogen, ozone, and repeated respiratory virus exposures that induce and/or augment reactive oxygen species (ROS) in the airways have been identified [6-8]. Cellular oxidative stress induced by ROS plays a fundamental role in inflammation through the activation of inflammatory signals which activate stress kinases such as ERK1/2, p38 and JNK, which in turn activate redox-sensitive transcription factors such as NF-κB that transcribe pro-inflammatory genes [9-12]. We have recently demonstrated that ROS-derived lipid aldehydes - glutathione (GSH) conjugates and their metabolic products are mediators of redox sensitive signaling. Aldose reductase (AR), an enzyme that reduces glucose to sorbitol in the polyol pathway, has been shown to efficiently reduce lipid aldehydes and their GSH conjugates (Km lipid aldehydes 10-30 μM and Km glucose ~50 mM) [13]. Further, we have shown that reduced product of lipid aldehyde-GSH conjugate could activate NF-κB and AP-1 via PLC/PKC/MAPK pathway [14,15]. Inhibition of AR significantly decreases the activation of these kinases and transcription factors, which results in decreased inflammation caused by various stimuli including high glucose, cytokines, growth factors, allergens and carcinogens.

Although, current regimen of asthma therapy, which includes corticosteroids in the form of inhalers, aims at reducing the inflammation, the attacks and/or exacerbation are not prevented. Various studies have demonstrated that antioxidants or ROS scavengers could reduce inflammation in experimental models, so do the inhibitors of the specific signaling kinases and could be critical targets in the amelioration of asthma [16-21]. However, these approaches have not resulted in the cure for asthma. Therefore, identification of novel targets and approaches to prevent or treat asthma is important. We have recently shown that AR could be a novel target to prevent or ameliorate inflammation and AR inhibitors could be developed as potential anti-inflammatory drugs [22,23]. Further, we have shown that inhibition or genetic ablation of AR could prevent allergen- or bacterial endotoxin-induced inflammation in human airway epithelial cells [24,25]. Similarly, administration of AR inhibitors to allergen-sensitized mice prior to allergen challenge prevented eosinophils infiltration in the lung, release of Th2 cytokines and chemokines and allergen associated hyper-responsiveness [24,25]. These results suggest that AR could be an important mediator of airway inflammation in allergic asthma and thus a novel molecular target to treat asthma. However, to unequivocally confirm that effects of AR inhibitors are specific to airway inflammation and not due to off-target effects, here we have used AR gene deletion approach by using AR null (AR^-/-^) mice and also examined the efficacy of a very potent and specific AR inhibitor, fidarestat [26,27], via enteral (oral) administration as opposed to parenteral in previous studies.

The results shown here demonstrate that RWE sensitization and challenge increased airway resistance, mucus hypersecretion, eosinophils infiltration and inflammatory cytokines and chemokines in wild-type (WT) mice and these changes were significantly decreased in AR^-/- ^mice. Further, oral administration of fidarestat efficiently and significantly decreased RWE-induced inflammatory cells infiltration and airway hyper-responsiveness and increased CD4^+^CD25^+^FoxP3^+ ^T regulatory cells (Tregs) population as compared to mice not treated with fidarestat. These results strongly support our hypothesis that AR is a key mediator of airway inflammation in RWE-induced allergic asthma model and suggest the effectiveness of oral AR inhibitor treatment for allergic asthma.

## Methods

### Animals

Approximately 6-8 weeks old Balb/c mice were purchased from Harlan Sprague-Dawley (San Diego, CA, USA) and used for experiments with the AR inhibitor. AR null (AR^-/-^) mice on C57BL/6 genetic background, were bred and maintained in a pathogen-free condition in the animal resource center at the University of Texas Medical Branch at Galveston (UTMB), TX, USA and were used for all knockout studies and showed no complications except for slightly increased urination [28]. The animals were maintained under the 12 h light and dark cycles, and were given food and water *ad libitum*. All animal experiments were performed according to the National Institutes of Health Guidelines for Care and Use of Experimental Animals and approved by the University of Texas Medical Branch Animal Care and Use Committee.

### Sensitization and challenge of animals

The mice were sensitized and challenged with RWE (Greer's laboratory; Lenoir, NC) as described by us previously [24]. Briefly, mice were sensitized with two intraperitoneal administrations of endotoxin-free RWE 150 μg/100 μl, combined with alum adjuvant in a 3:1 ratio, on days 0 and 4. In fidarestat (obtained as a gift chemical from Sanwa Kagaku Kenkyusho Co. Ltd., Nagoya, Japan) -treated groups, animals received the drug either by gavage (b.i.d.) or in drinking water *ad-libitum *so that they received a total of 200 μg fidarestat per day (estimated; based upon daily water consumption per mouse) starting on day 9. On day 11, mice (*n *= 6) were challenged intranasally with RWE (100 μg) dissolved in 50 μl of PBS. Control groups of mice were challenged with equivalent volumes of PBS.

### BAL cytology

To evaluate inflammation, animals from all experimental groups were euthanized on day 14 with ketamine (135 mg/kg body wt) and xylazine (15 mg/kg body wt), and the lungs were lavaged with two 0.8 ml aliquots of ice-cold PBS. The cells were collected by centrifugation (1000 *g*, for 10 min at 4°C), re-suspended in one ml PBS and total cell counts were determined [24,25]. The differential cell count was performed on cytocentrifuge preparations stained with hematoxylin and eosin. The cell composition in the BAL was evaluated by a pathologist, blinded to treatment groups, to obtain data for each lung. The representative fields were photographed with a Photometrix CoolSNAP Fx camera mounted on a NIKON Eclipse TE 200 UV microscope.

### Histo-pathology of the lungs

The lungs were lavaged with and fixed in 4% buffered-formaldehyde followed by paraffin embedding and sectioning to 5 μm thickness at our histology core facility (Research histology Core, UTMB, Galveston). The histo-pathological examination was performed on the H&E stained sections using a Photometrix CoolSNAP Fx camera mounted on a NIKON Eclipse TE 200 UV microscope. The representative fields were photographed and presented (n = 6).

### Periodic Acid Schiff (PAS) Staining

Metaplasia of the lung epithelial cells was determined by PAS-staining of formalin-fixed, paraffin-embedded lung sections [29]. The stained sections were observed as above and representative fields were photographed with a Photometrix CoolSNAP Fx camera mounted on a NIKON Eclipse TE 200 UV microscope

### Determination of Mucin by ELISA

Muc5A/C level in the BAL was determined by ELISA using commercially available mouse anti-Muc5A/C ELISA essentially as described by the manufacturer (Cosmo Bio USA; Carlsbad, CA).

### Determine of airway hyper-responsiveness

Airway responsiveness was measured in unrestrained, conscious mice 3 days after the RWE-challenge [24]. The mice were placed in a barometric plethysmographic chamber, baseline readings were taken and averaged for 3 min. Aerosolized methacholine in increasing concentrations (from 10 to 100 mg/ml) were nebulized through an inlet of the main chamber for 3 min. Readings were taken and averaged for 3 min after each nebulization and PENH was determined. PENH was calculated as (expiratory time/relaxation time^-1^) × (peak expiratory flow/peak inspiratory flow) according to the manufacturers' protocol. PENH correlates with pulmonary airflow resistance or obstruction and was used as a measure of airway responsiveness to methacholine.

### Immunofluorescence staining and FACS analysis

Anti mouse Tregs detection Kit (Cat# 130-094-164) from Miltenyi biotech (Auburn, CA) was used to determine the percentage of FoxP3^+ ^cells in CD4^+^/CD25^+ ^T cell population. The mouse spleenocytes were isolated and first surface stained with FITC-labeled anti-mouse CD4 and PE-labeled anti-mouse CD25 mAb per standard protocol. These cells were subsequently stained with APC-labeled antimouse-FoxP3 mAb or rat IgG2a nonspecific isotype control mAb, according to the manufacturer's recommendations (Miltenyi Biotech) and hundred thousand events from each sample were analyzed by flow cytometry using the LYSIS II software (FACScan, BD Pharmingen) and presented as the percent population of either CD4^+^/FoxP3^+ ^or CD25^+^/FoxP3^+ ^T cells.

### Statistical analysis

The data presented are mean ± SD and *p *values were determined by unpaired, two-tailed Student's t test and analyzed by ANOVA. The *p *values less than 0.05 were considered statistically significant.

## Results

### AR deficiency protects against RWE-induced airway hyper-responsiveness in mice

**Figure 1:**
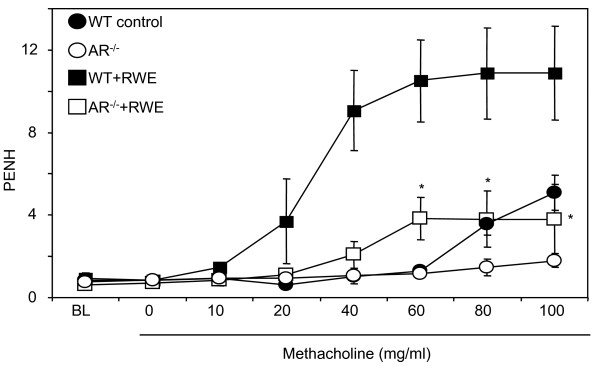
**AR deficiency prevents methacholine-induced airway hyper-responsiveness in RWE-challenged mice**. The changes in PENH were measured by whole-body plethysmography. Mice were placed in a barometric plethysmographic chamber and PENH, an index of airway obstruction, was determined and plotted against the increasing concentration of methacholine. Each data point represents mean±SD of 4 mice for each group. *p < 0.01 vs. WT-RWE. RWE, ragweed pollen extract; WT, wild-type; BL, base line; PENH, enhanced pause.

Since inflammatory changes in allergic asthma lead to blockage of airway passages and cause airway resistance leading to hyper-responsiveness, we first subjected the RWE-sensitized and challenged mice to whole body unrestrained plethysmography and quantitatively measured airway responsiveness in response to methacholine inhalation. As shown in Figure 1, in WT mice, PENH elevated dose-dependently in response to methacholine challenge as compared to control mice treated with PBS alone, whereas in AR^-/- ^mice sensitized and challenged with RWE, the PENH values were significantly (~70%; p < 0.01) less compared to RWE-sensitized and challenged WT mice. These results indicate that deletion of AR significantly prevented the patho-physiological effects of allergic asthma in mice.

### AR deficiency protects against RWE-induced mucous cell metaplasia and mucin hyper-secretion in mice lung

**Figure 2:**
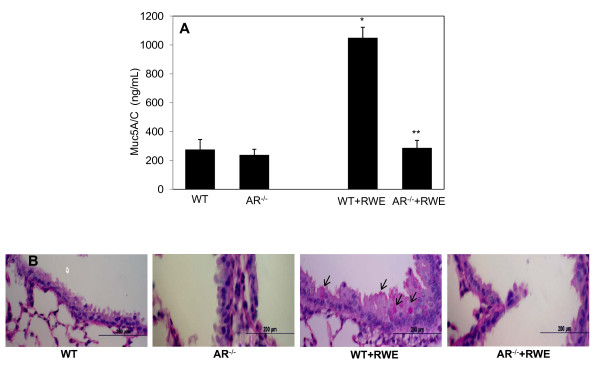
**AR deficiency decreases mucin levels and mucous cell metaplasia in airway of RWE-challenged mice**. (A) RWE-sensitized WT and AR^-/- ^mice were challenged with RWE and 72 h later the lungs were lavaged with cold PBS. The supernantant was used for the determination of Mucin5A/C using mouse Muc5A/c specific ELISA. The bars represent mean±SD (n = 4) *p = 0.00068 vs. WT Control; **p = 0.00027 vs. WT-RWE. (B) The lungs were harvested, perfused and fixed with 4% paraformaldehyde and embedded in paraffin. The sections were stained with PAS stain and observed under light microscope and photomicrographs were acquired. A representative photomicrograph from each group is shown (Magnification 200×) (n = 4). RWE, ragweed pollen extract; WT, wild-type; AR^-/-^, aldose reductase null mice.

Excessive mucus secretion is known to cause airway obstruction, which is one of the main patho-physiological characteristic of allergic asthma. Inhibition of mucous cell metaplasia and subsequent mucin hypersecretion could minimize allergic attacks and exacerbation in susceptible individuals [30,31]. Therefore, to investigate if AR deficiency affects the mucin levels in mice, we measured the levels of Muc5A/C in the BAL fluid by ELISA. We found that mucin levels increased approximately 4-folds in RWE-challenged WT mice as compared to WT control, whereas in BAL of RWE-challenged AR^-/- ^mice the levels of Muc5A/C were significantly (p < 0.0003) less (Figure 2A). Similarly, PAS-staining of the lung sections showed increased number of PAS positive cells in WT mice than in AR^-/- ^mice (Figure 2B).

### AR deficiency protects against RWE-induced infiltration of eosinophils in mice lung

**Figure 3:**
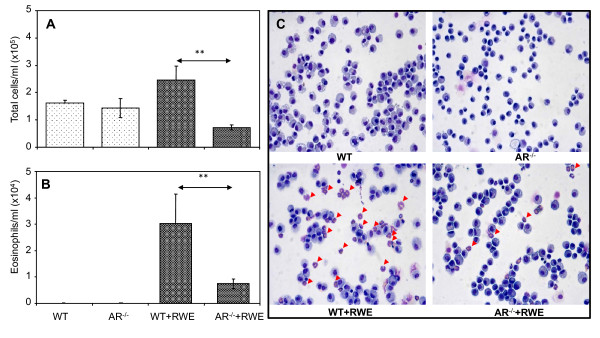
**AR deficiency prevents accumulation of eosinophils in BAL of RWE-challenged mice**. The mice were sensitized and challenged with PBS or RWE and 72 h later lungs were lavaged and differential cell counts were determined as described in methods. The total cells (*A*) and eosinophils (*B*) per ml BAL fluid are shown as bar diagram. The bars represent mean ± SD (*n = 4*-6). **p <0.001. (C) The photomicrograph showing a representative field of H&E stained preparations from each group of mice (Magnification 200×). Arrowheads point to eosinophils in BAL cytospin preparation (n = 4-6). RWE, ragweed pollen extract; WT, wild-type; AR^-/-^, aldose reductase null mice.

**Figure 4:**
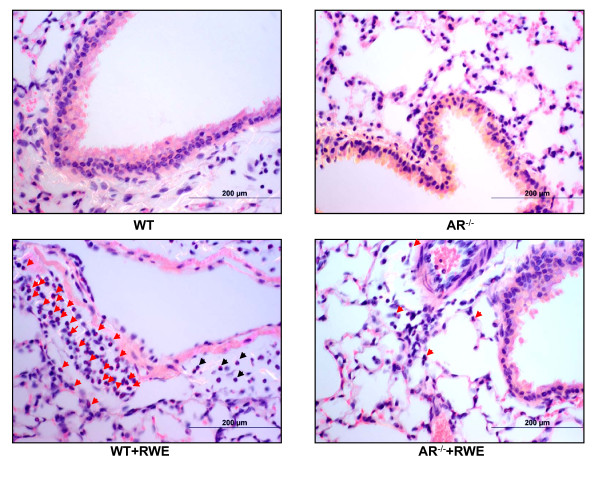
**AR deficiency blocks inflammatory cells infiltration in RWE-challenged mice lung**. Lungs of RWE-sensitized and challenged mice were lavaged and fixed. The fixed lungs were sectioned and stained with H&E. Arrowheads indicate sites of inflammatory cells infiltration induced by RWE-challenge (n = 4-6). RWE, ragweed pollen extract; WT, wild-type; AR^-/-^, aldose reductase null mice.

Accumulation of inflammatory cells in BAL fluid and infiltration into lung sub-epithelial spaces in response to allergens is indicative of airway inflammation. Since pathological responses of RWE-challenge i.e. airway resistance and mucus secretion were significantly prevented in AR^-/- ^mice, we next examined the effect of AR deficiency on eosinophils infiltration, main characteristic feature of allergic asthma, in the BAL fluid as well as in the lung. As shown in Figure 3A, and 3B there was a significant increase in the number of total cells as well as Eosinophils in the BAL fluid from the RWE-sensitized and -challenged WT mice whereas the number was significantly less in the BAL of RWE-sensitized and -challenged AR^-/- ^mice. The eosinophils in the BAL were approximately 21% in WT mice when sensitized and challenged with RWE, whereas in AR^-/- ^mice significantly less number of eosinophils (~9%) were observed as compared to 0% in both, WT and AR^-/-^, control mice. Further, the percentages of neutrophils in the BAL of RWE-sensitized and -challenged WT and AR^-/- ^mice were 0.33 and 0.13 respectively, while those of lymphocytes were 0.19 and 0.15, respectively. Similarly, when BAL cytospin preparations were compared, there were more eosinophils in the RWE-challenged WT mice than in RWE-challenged AR^-/- ^mice (Figure 3C). Further, peri-vascular and peri-bronchial inflammation, as determined by infiltration of eosinophils in these regions of the lung tissue, in the RWE-challenged AR^-/- ^mice was markedly less compared to RWE-challenged WT mice (Figure 4). These results indicate protective role of AR deficiency in the RWE-induced inflammation in mice lung.

### AR deficiency protects from RWE-induced secretion of cytokines and chemokines in mice lung

Th2 type cytokines and chemokines play an important role in the allergic airway disease therefore, we determined the effect of AR deficiency on the RWE-induced expression of Th2 type cytokines and chemokines in the BAL fluid using multiplex cytokine and chemokines assay. As shown in Table 1, RWE-challenge of the WT mice resulted in increased levels of various cytokines and chemokines, especially the levels of Th2 cytokines IL-4, IL-5, IL-6, IL-7, IL-10 and IL-13, and chemokines such as G-CSF, IP-10, MIP-1, and KC were significantly elevated in the WT mice BAL fluid upon RWE challenge. The levels of these inflammatory markers were significantly lower in RWE-challenged AR^-/- ^mice BAL fluid. In WT as well as AR^-/- ^control mice there was no significant alteration in the level of these cytokines and chemokines.

**Table 1:** AR deficiency decreases cytokines and chemokines levels in the lungs of RWE-challenged mice.

*Cytokine and chemokines*	*WT Control*	*AR^-/- ^Control*	*WT-RWE*	*AR^-/-^-RWE*
**1. IL-1a**	1.1 ± 0.5	1.6 ± 1.0	35.2 ± 1.9***	22.0 ± 3.3^#^
**2. IL-2**	11.0 ± 0.8	12.4 ± 0.7	19.7 ± 3.1*	12.3 ± 2.0^#^
**3. IL-4**	ND^¶^	ND^¶^	8.9 ± 0.8*	1.5 ± 0.7^#^
**4. IL-5**	1.6 ± 1.6	6.5 ± 1.5	108.5 ± 18.7**	53.0 ± 4.0^#^
**5. IL-6**	2.4 ± 1.3	3.2 ± 0.4	67.5 ± 12.1**	27.4 ± 2.4^#^
**6. IL-7**	2.4 ± 1.4	2.4 ± 1.3	10.1 ± 0.7**	5.3 ± 0.2^#^
**7. IL-10**	2.1 ± 1.8	1.6 ± 1.6	24.0 ± 3.6**	9.7 ± 4.2^#^
**8. IL-13**	2.4 ± 1.3	3.2 ± 0.5	76.6 ± 6.5*	15.5 ± 2.3^#^
**9. G-CSF**	9.2 ± 1.8	9.5 ± 3.9	842.8 ± 115.4**	239.4 ± 56.0^##^
**10. IP-10**	4.0 ± 1.4	4.8 ± 2.1	79.9 ± 14.8**	24.3 ± 3.9^#^
**11. MIP-1**	ND^¶^	0.05 ±0.03	46.3 ± 2.7***	24.5 ± 0.5^##^
**12. KC**	2.7 ± 1.7	2.8 ± 1.6	33.1 ± 1.8**	15.6 ± 1.65 ^##^
**13. RANTES**	0.9 ± 0.1	1.1 ± 0.7	4.4 ± 1.1*	1.9 ± 0.4^#^

The levels of cytokines and chemokines in the BAL fluids were measured using the magnetic beads-based multiplex cytokine assay system. Data (pico-gm/mL) are presented as means ± SD (n = 4).

*p < 0.02 vs. WT control mice; **p < 0.008 vs. WT control mice; ***p<0.0002 vs. unchallenged WT control mice; ^#^*p*<0.03 vs. WT-RWE-challenged mice, ^##^*p*<0.005 vs. WT-RWE-challenged mice. ^¶^ND, non-detectable

### Oral administration of AR inhibitor, fidarestat, prevented RWE-induced eosinophils infiltration and hyper-reactivity in mice airway

**Figure 5:**
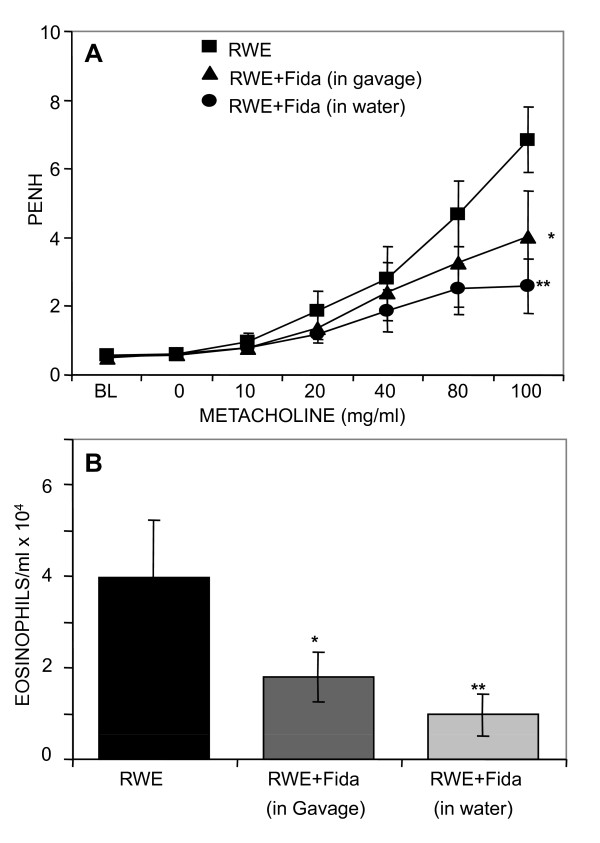
**AR inhibition by oral administration of fidarestat blocks airway hyper-responsiveness and accumulation of inflammatory cells in BAL of RWE-challenged mice**. (*A*) The changes in PENH were measured by whole-body plethysmography. Mice were placed in a barometric plethysmographic chamber and PENH was determined and plotted against the increasing concentration of methacholine. Each data point represents mean±SD (n = 6). *p < 0.05 and **p <0.01 vs. RWE, (B) Eosinophils in the BAL fluid were determined 72 h after RWE-challenge and presented as bar diagram. The bars represent (mean ± SD) eosinophils per ml BAL fluid (*n = *6). *p < 0.05 and **p <0.01 vs. RWE. BL, baseline; RWE, ragweed pollen extract; Fida, fidarestat. PENH, enhanced pause.

Next, we tested the efficacy of orally administered, very potent and specific AR inhibitor, fidarestat, in RWE-induced asthma in mice. The inhibitor was administered through oral route either by gavage (b.i.d.) or in drinking water *ad-libitum *starting 24 h before challenge and continued until the end of experiment (200 μg fidarestat per kg body weight per day). As shown in Figure 5A airway hyper reactivity in RWE-challenged mice significantly decreased when mice were administered fidarestat in water *ad-libitum *or by gavage as compared to RWE-challenged mice not given fidarestat. Similar pattern was observed when eosinophils accumulation in BAL fluid was determined (Figure 5B). Fidarestat administration did not change the basal airway hyper reactivity in control mice not sensitized and challenged with RWE (data not included).

**Figure 6:**
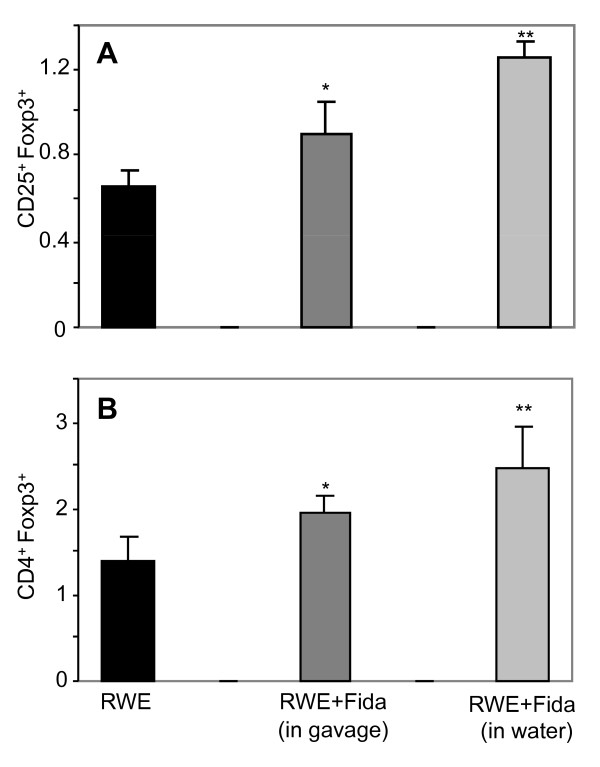
**Oral administration of fidarestat increased the percentage of FoxP3^+ ^Tregs in the spleen-derived CD4^+^CD25^+ ^T cells population following RWE-challenge**. The spleen-derived CD4^+^CD25^+ ^T-cells from different groups were stained with FoxP3 antibodies and subjected to FACS analysis. The bars represent (A) CD25^+^FoxP3^+ ^and (B) CD4^+^FoxP3^+ ^population, data expressed as mean±SD (n = 5), *p<0.05 vs. RWE; **p<0.01 vs. RWE. RWE, ragweed pollen extract, Fida, fidarestat.

Further, various studies have implicated up-regulation of Tregs in the amelioration of asthma pathogenesis (32-34). Therefore, we examined whether AR inhibitor affects the Tregs population in the spleen of RWE-sensitized and challenged mice. We analyzed the population of Tregs (CD4^+^/FoxP3^+ ^or CD25^+^/FoxP3^+^) in spleen-derived T cells by FACS analysis and found that fidarestat-treatment of RWE-challenged mice led to elevated levels of Tregs compared to that of RWE-challenge alone (Figure 6). Taken together, these findings suggest that AR inhibition could significantly decrease patho-physiological changes as it leads to increased percentage of Tregs in the experimental murine model of RWE-induced asthma.

## Discussion

In the present study, we have used AR^-/- ^mice to investigate the effect of AR deficiency on RWE-induced airway inflammation. Our results suggest that while in WT mice RWE-sensitization and -challenge resulted in increased inflammation as determined by accumulation of eosinophils in the lungs and BAL fluid, increased cytokines and chemokines levels and a dose-dependent increase in PENH, the AR^-/- ^mice showed significantly less inflammation as they had decreased levels of eosinophils, cytokines and chemokines and airway hyper-responsiveness. Further, oral administration of fidarestat, significantly decreased the number of eosinophils and hyper-responsiveness to methacholine and elevated the levels of Tregs compared to mice sensitized and challenged with RWE alone. Although it is premature to speculate the therapeutic potential of AR inhibitors in allergic asthma, our present results in mice besides strongly supporting our earlier results, further suggest that AR inhibitors such as fidarestat could be preventive as well as therapeutic in allergic asthma.

The pathophysiology of asthma is a multi-cellular event involving airway epithelium, lung fibroblasts and smooth muscles, resident macrophages and dendritic cells and infiltrated leukocytes including eosinophils, neutrophils, and polarized Th2 cells. Besides the fact that allergic asthma is Th2-mediated pathology, airway epithelium plays an important role as it gets exposed to the external allergens first and starts an inflammatory event that gets further accentuated by other cell types [35]. The airway epithelium in asthmatics becomes fragile and defective in repair and antioxidant functions that lead to compromised tight junctions, which results in greater destruction and loss of epithelial cells leading to enhanced airway responsiveness. Our results in the present study demonstrate that AR^-/- ^mice showed significantly decreased airway responsiveness which is in concert with our earlier findings that AR inhibition prevents allergen-induced epithelial cell loss due to apoptosis [24]. Further, decrease in airway responsiveness could also result from significantly decreased goblet cell metaplasia in AR^-/- ^mice airway subsequent to RWE-challenge (Figure 2), which is in concert with our earlier observations [29].

Increased oxidative stress due to release of ROS from cells upon exposure to the oxidants including allergens, bacterial/viral antigens, cigarette smoke and vehicle exhaust and particulate matters as well as from the inflammatory and immune cells in the airway has been demonstrated [9,36-39]. Increased ROS levels in airway epithelial cells upon allergen exposure activate redox-sensitive signaling cascade leading to the activation of transcription factors such as NF-κB and AP-1 which transcribe various inflammatory mediator genes and promote apoptosis [24,25]. Further, release of inflammatory markers results in epithelial cell loss as well as chemo-attraction of leukocytes such as mast cells, basophiles, Eosinophils, neutrophils, T-cells, and macrophages. The activated inflammatory cells further enhance oxidative stress by directly releasing ROS such as superoxides and H_2_O_2 _and in addition release inflammatory cytokines, especially Th2 cytokines that lead to pathogenesis of allergic asthma. In our study, a significant decrease in total inflammatory cells, more specifically that of eosinophils in BAL and lung sub-epithelium of AR^-/- ^mice (Figure 3, and 4) indicates that AR-deficiency results in suppression of allergen-induced activation of airway epithelium leading to subsequent decrease in inflammatory cells infiltration and release of inflammatory cytokines and chemokines in BAL (Table 1). These evidences thus strongly suggest the role of AR in the pathogenesis of allergic asthma.

Subsequent to confirming that AR is indeed involved in the mediation of allergic asthma, we tested the oral efficacy of a highly specific and potent AR inhibitor, fidarestat, which was administered either by gavage or in drinking water *ad-libitum*. A number of AR inhibitors including sorbinil, tolrestat, and zenarestat have undergone clinical trials for diabetic complications and were found to have several irreversible side-effects [40]. Fidarestat, on the other hand, has undergone phase III clinical trial for diabetic neuropathy and found to have no irreversible side effects [41]. The findings in Figure 5 indicate that fidarestat when given to mice orally significantly prevented airway hyper-responsiveness and eosinophils infiltration in response to RWE-sensitization and challenge.

Several investigators have implicated up-regulation of Tregs in the amelioration of asthma pathogenesis [32,33]. McGee and Agrawal (2009) have demonstrated that Tregs derived from lung and spleen when transferred to mice sensitized and challenged with cockroach antigen, they completely reversed airway hyper-responsiveness and allergic airway inflammation and this effect was long lasting [34], suggesting protective role of Tregs in allergic asthma. Therefore, we examined the affect of AR inhibition on Tregs population in the spleen of RWE-sensitized and -challenged mice. To our surprise, we observed that fidarestat-treatment of RWE-challenged mice led to elevated population of Tregs (CD4^+^/FoxP3^+ ^or CD25^+^/FoxP3^+^) in spleen-derived CD4^+^/CD25^+ ^T cells pool as compared to RWE alone -challenged mice (Figure 6). Although these results indicate that inhibition of AR increased the percentage of Tregs in spleen which could modify the RWE-induced airway hyper-responsiveness and inflammation (Figures 5A and 5B), the mechanism of such upregulation of Tregs by AR inhibition is still unclear.

**Figure 7:**
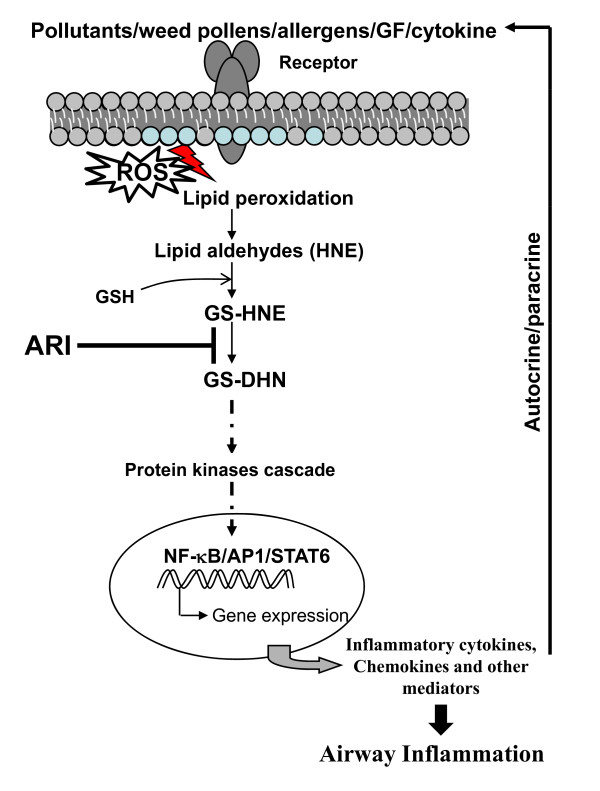
**Schematic of working mechanism of AR in airway inflammation**. The ROS generated by different stimuli including pollens, allergens, growth factors and cytokines are known oxidize membrane lipids to lipid aldehydes such as 4-hydroxynonenal (HNE), which could readily conjugate with glutathione (GSH) and form GS-lipid aldehydes (e.g. GS-HNE). AR reduces GS-HNE to GS-DHN which activates PLC, PKC and PI3K which in turn could lead to activation of transcription factors including NF-κB, AP-1, and STAT-6 that transcribe various inflammatory genes. The inflammatory markers propagate the signals in autocrine and/or paracrine fashion and cause airway inflammation and asthma pathogenesis. AR inhibition blocks this cascade upstream of various kinases and thus prevents inflammation.

Our recent studies have shown that AR, an enzyme that was contemplated to reduce aldehyde-sugars such as glucose to corresponding alcohol such as sorbitol and cause diabetic complications including diabetic neuropathy, retinopathy and nephropathy, in fact more efficiently catalyzes the reduction of lipid peroxidation-generated lipid derived-aldehydes such as 4-hydroxynonenal (HNE) (K_m _HNE: 10-30 μM Vs. K_m _glucose: 50 mM) [13,42]. HNE and their glutathione conjugates, GS-HNE are reduced by AR to corresponding alcohols such as dihydroxynonene (DHN) or glutathiolated- dihydroxynonene (GS-DHN) [42]. It is well known that cytokines, chemokines, growth factors, endotoxins, allergens and environmental pollutants cause increased ROS generation in the biological system, which could oxidize cellular biomolecules. Membrane lipids, when oxidized by ROS, form lipid peroxidation products, such as HNE which are considered as the biomarkers in the oxidative stress-induced inflammatory diseases. Increased levels of HNE, found in the asthmatics lungs, could readily react with cellular glutathione and form GS-HNE conjugates leading to decrease in reduced glutathione. Decrease in the levels of GSH along with increased GSSG in BAL fluid has been reported in asthmatic children and found to be associated with increased malondialdehyde, 8-isoprostanes, 4-HNE and H_2_O_2 _[43]. It has been shown that under inflammatory conditions the expression of AR increases significantly and this increase in expression is not related to increased glucose levels [44,45]. Thus, low K_m _of AR for lipid-aldehydes and their GSH-conjugates and oxidative stress-induced and glucose-independent increased expression of AR suggest that AR plays a significant role in oxidative stress-induced inflammation besides being an aldehyde detoxifying enzyme. We have demonstrated that AR-catalyzed reduced product of GSH-lipid aldehydes i.e. GS-DHN mediates the activation of redox-sensitive transcription factors NF-κB and AP1 via PLC/PKC/IKK/MAPK pathway that causes inflammation [14,15], thus inhibition or deficiency of AR should block the inflammatory signals (Figure 7). Therefore, our results present a novel evidence that deficiency of AR significantly decreases inflammatory and pathological changes upon allergen challenge in mice which could be due to lack of AR-catalyzed mediator of inflammatory signals in AR^-/- ^mice.

## Conclusions

The present study, by utilizing a knockout animal model, confirms the role of AR in the mediation of allergic airway inflammation in mice and also suggests that oral administration of AR inhibitor, fidarestat, could be effective in preventing allergen-induced inflammatory changes.

## List of Abbreviations

AR: aldose reductase; RWE: ragweed pollen extract; WT: wild type; BAL: bronchoalveolar lavage; ROS: reactive oxygen species; AR^-/- ^mice: AR deficient mice; GSH: glutathione; PLC: phospholipase C; PKC: protein kinase C; MAPK: mitogen activated protein kinases; NF-κB: nuclear factor kappa B; AP1: activated protein 1; PENH: enhanced pause; PAS: periodic acid Schiff; Muc5A/C: mucin 5A/C; Tregs: T regulatory cells; HNE: 4-hydroxynonenal; GS-DHN: glutathiolated- dihydroxynonene; GS-HNE: glutathiolated 4hydroxynonenal; GSSG: oxidized glutathione.

## Competing interests

The authors declare that they have no competing interests.

## Authors' contributions

UCSY and LAA carried out the experimental work and performed the statistical analysis. UCSY participated in the design of the study and drafted the manuscript. SKS, KVR and IB conceived the idea, and participated in its design and coordination and draft of the manuscript. All authors read and approved the final manuscript.
